# Supplementary material for: Precision multiparameter tracking of inflammation on timescales of hours to years using serial dried blood spots
Source: Bioanalysis. 2020 Apr 7:10.4155/bio-2019-0278. doi: 10.4155/bio-2019-0278 (PMC7372997; doi:10.4155/bio-2019-0278)
Supplement: Supplementary file 1 [file bio-2019-0278-s1.docx]

**Supplementary information for**

**Precision Multiparameter Tracking of Inflammation on Timescales of Hours to Years Using Serial Dried Blood Spots**

Details of datasets:

Dataset A results have been previously described in part [1] and were obtained using a liquid chromatography tandem mass spectrometry (LC-MS/MS) platform consisting of an Agilent 1290 LC system operating at 600 μL/min and an Agilent 6490 triple quadrupole MS. Replicate standards were prepared as punches of DBS (4 punches of 1.5 mm diameter filter paper were used per well). Datasets B and C employed an Eksigent microflow LC system operating at 10 μL/min connected to a Sciex 6500 Q-trap MS. Dataset B (436 samples from four subjects) focused on measurements of a panel of gastrointestinal inflammation related proteins. Replicate standards were prepared by pipetting whole blood onto pre-cut 6 mm diameter disks of Whatman 903 filter paper in sample wells. Dataset C was comprised of two sequential panels applied to the same DBS digests.

Nine peptides were measured in all datasets (including Alb, Hx, and IgM) and 12 were measured in both Datasets A, C and D. As part of our continuing efforts to increase assay precision, the 27-plex used in Dataset C was measured as two sequential SISCAPA multiplex subpanels, both of which included the normalizing proteins albumin (Alb), hemopexin (Hx) and total immunoglobulin M (IgM). Datasets A and B were obtained using a single MRM per peptide (measured as a single multiplex panel), while Dataset C used the average of 1 to 3 of the best quality MRM transitions available for the respective peptides. Datasets B and C were each merged with Dataset A using a run-to-run scale factor for each protein in common between datasets in order to account for potential shifts in the absolute amounts of the SIS peptides during storage between the analytical runs. The scale factor was derived from the ratio of average values for a protein in subject samples that were analyzed in both Datasets (88 samples analyzed in both Datasets A and B, and 19 samples analyzed in both Datasets B and C, in each case using duplicate punches from the same DBS card). When samples from the same subject were shared between datasets this ratio was subject-specific; for subjects without shared samples an average of the subject-specific ratios was used.

PAR measurements in Datasets A and D were determined using MassHunter Quantitative Analysis (v. B.05.02), while in Datasets B and C the Sciex MultiQuant program (v. 3.0.2) was used. SIS peptides were labeled with 13C, 15N lysine or arginine as the c-terminal amino acid.

CVs were typically somewhat higher in Datasets A and B, which were analyzed earlier in the process of assay optimization. In Dataset C the CV over all 26 peptides measured in two successive multiplex enrichments from 4 replicate dried blood standards in each of nine 96-well plates (72 measurements) was reduced from 10.2% prior to volume normalization to 4.6% after normalization (Supplementary Table 1). Similarly, the overall average CV in Dataset A was reduced from 9.4% to 7.4% by normalization. In Dataset B, normalization had little effect (6.5% vs 6.1%) because in this case the standards were prepared by drying the same measured volume of whole blood onto pre-cut filter paper disks in wells, such that there was little or no volume variation to correct. In Dataset C, key inflammation markers such as C-reactive protein (CRP), serum amyloid A (SAA) and lipopolysaccharide binding protein (LPSBP) showed normalized CVs in the standard samples of 3.3% - 3.7%.

Alternative multi-parameter indices of inflammatory effects

In the past, efforts to move beyond single point CRP measurements have explored two scenarios: a multi-protein ratio at one time point, or alternatively a single protein measured at multiple time points. Both approaches have significant limitations.

In the first scenario, several ratios involving CRP have been explored for clinical diagnostic applications, including, for example, the ratio of SAA to CRP, proposed as an indicator of infectious disease severity in children [2]. In our results this ratio does appear to distinguish between large and small infection responses when measured at the peak of response (Fig 5C); however, the ratio changes so rapidly during an infection (up to 20-fold change over the course of a few days as shown in Fig 2B) that sampling at any point off-peak can generate an incorrect prediction. Likewise the ratio of fibrinogen to CRP has generated equivocal results [3–5] since it also varies in time due to the difference in expression time courses for the two proteins. Somewhat more success has been achieved using the ratio of CRP to albumin as a long-term outcome predictor in pancreatic cancer [6], surgery in the elderly [7], sepsis [8], and stent restenosis [9], while a similar 3-level index combining CRP and Alb measurements (the Glasgow Prognostic Score [10]; GPS) has achieved more widespread use as an outcome predictor in many cancers. The greater success of the CRP/Alb ratio and GPS may be attributed to the fact that the components move in opposite directions during the APR (being positive and negative APR respectively), so that inflammation-driven change of either protein from baseline drives the ratio upwards. Nevertheless, the fact that individual APR proteins follow different time courses, making any ratio between proteins time-varying, substantially limits the utility of protein:protein ratios at any single timepoint.

The second scenario, using an index of CRP changes over time, has been more generally successful for evaluating infection severity [11], risk of death in intensive care [12], presence of post-surgical infection [13] and likelihood of success or failure of treatment in community-acquired pneumonia [14], sepsis [15], Crohn’s disease [16] and stem cell transplantation [17]. These methods make use of changes occurring between sample timepoints and thus provide more information than single point CRP measurements. Nevertheless, because of the fast-response nature of CRP, they do not incorporate information on inflammation levels outside narrow time-windows bracketing sample collection, nor, without effective prior measurement of personal baseline levels, can they provide a personalized indication of inflammation severity.

Supplementary References

1. Razavi M, Anderson NL, Yip R, Pope ME, Pearson TW. Multiplexed longitudinal measurement of protein biomarkers in DBS using an automated SISCAPA workflow. *Bioanalysis*. 8(15), 1597–1609 (2016).

2. Huttunen T, Teppo A, Lupisan S, Ruutu P, Nohynek H. Correlation Between the Severity of Infectious Diseases in Children and the Ratio of Serum Amyloid A Protein and C-reactive Protein. *Scand. J. Infect. Dis.* 35(8), 488–490 (2003).

3. Windsperger K, Lehner R. The fibrinogen/CRP ratio as a new parameter for the diagnosis of disseminated intravascular coagulation in patients with HELLP syndrome and as a predictive factor for neonatal outcome. *Am. J. Obstet. Gynecol.* 208(2), 118.e1-118.e7 (2013).

4. Brucka-Kaczor A, Woźniak P, Litwińska E, Pięta-Dolińska A, Oszukowski P. Maternal and fetal parameters including umbilical artery PI and fibrinogen/CRP ratio as predictive factors of perinatal outcome in women with HELLP syndrome. *Pol. Gynaecol.* 86(3), 176–181 (2015).

5. Winder T, Posch F, Asamer E, *et al.* An elevated fibrinogen/CRP ratio predicts a remarkable survival advantage in patients with metastatic pancreatic cancer. *Ann. Oncol.* [Internet]. 27(suppl_6) (2016). Available from: DOI: 10.1093/annonc/mdw371.77.

6. Liu Z, Jin K, Guo M, *et al.* Prognostic Value of the CRP/Alb Ratio, a Novel Inflammation-Based Score in Pancreatic Cancer. *Ann. Surg. Oncol.* 24(2), 561–568 (2017).

7. Simpson G, Saunders R, Wilson J, Magee C. The role of the neutrophil:lymphocyte ratio (NLR) and the CRP:albumin ratio (CAR) in predicting mortality following emergency laparotomy in the over 80 age group. *Eur. J. Trauma Emerg. Surg.* 44(6), 877–882 (2018).

8. Kim MH, Ahn JY, Song JE, *et al.* The C-Reactive Protein/Albumin Ratio as an Independent Predictor of Mortality in Patients with Severe Sepsis or Septic Shock Treated with Early Goal-Directed Therapy. *PLOS ONE*. 10(7), e0132109 (2015).

9. Aksu U, Gulcu O, Aksakal E, *et al.* The association between CRP / Albumin ratio and in‐stent restenosis development in patients with ST‐segment elevation myocardial infarction. *J. Clin. Lab. Anal.* 33(4), e22848 (2019).

10. McMillan DC. The systemic inflammation-based Glasgow Prognostic Score: A decade of experience in patients with cancer. *Cancer Treat. Rev.* 39(5), 534–540 (2013).

11. Peltola H, Jaakkola M. C-reactive Protein as a Serial Index of Severity. *Clin. Pediatr. (Phila.)*. 27(11), 532–537 (1988).

12. Coelho LM, Salluh JI, Soares M, *et al.* Patterns of c-reactive protein RATIO response in severe community-acquired pneumonia: a cohort study. *Crit. Care*. 16(2), R53 (2012).

13. Santonocito C, De Loecker I, Donadello K, *et al.* C-Reactive Protein Kinetics After Major Surgery: *Anesth. Analg.* 119(3), 624–629 (2014).

14. Pereira JM, Laszczyńska O, Azevedo A, *et al.* Early prediction of treatment failure in severe community-acquired pneumonia: The PRoFeSs score. *J. Crit. Care*. 53, 38–45 (2019).

15. Gutiérrez-Gutiérrez B, Morales I, Pérez-Galera S, *et al.* Predictive value of the kinetics of procalcitonin and C-reactive protein for early clinical stability in patients with bloodstream infections due to Gram-negative bacteria. *Diagn. Microbiol. Infect. Dis.* 93(1), 63–68 (2019).

16. Reinisch W, Wang Y, Oddens BJ, Link R. C‐reactive protein, an indicator for maintained response or remission to infliximab in patients with Crohn’s disease: a post‐hoc analysis from ACCENT I. *Aliment. Pharmacol. Ther.* 35(5), 568–576 (2012).

17. Fassas A, Miceli MH, Grazzlutti M, Dong L. Serial measurement of serum C-reactive protein levels can identify patients at risk for severe complications following autologous stem cell transplantation. *Leuk. Lymphoma*. 46(8), 1159–1161 (2005).

**Supplementary Figure legends:**

Supplementary Fig. 1. A) Amounts (fmol of peptide) for 9 proteins measured in 255 longitudinal DBS samples from subject S-04 before (top panel) or after (lower panel) DBS volume normalization by Alb, Hx, IgM. B) Amounts of 12 proteins mrasured in 411 serial samples from subject S-18 after normalization and division by personal baseline values.

Supplementary Fig 2. Whiskerplots of the amounts of 12 proteins in the baseline samples of each of 8 subjects. Protein amount has been normalized between proteins by dividing by the median amount in all subjects.

Supplementary Fig 3. Values for MPO normalized by personal baseline showing several infection events in which MPO temporarily increased.

Supplementary Fig 4. Values for IgM normalized by personal baseline showing two infection events (S-10 E11 and S-18 E6) in which IgM temporarily increased by more than 50% and the returned to baseline levels. A third event (S-01 E7) showed a temporary increase of about 30%.

Supplementary Table 1

|  | Dataset A Standards | | Dataset B Standards | | Dataset C Standards | |
| --- | --- | --- | --- | --- | --- | --- |
| Protein | CV Before Vol Normalization | CV After Vol Normalization | CV Before Vol Normalization | CV After Vol Normalization | CV Before Vol Normalization | CV After Vol Normalization |
| A1AG | 11.3% | 8.6% |  |  | 9.2% | 4.7% |
| Alb | 7.2% | 1.9% | 7.2% | 5.7% | 10.4% | 1.9% |
| C3 | 6.2% | 2.3% |  |  | 9.3% | 5.5% |
| CRP | 14.1% | 13.1% | 6.8% | 6.8% | 9.8% | 3.3% |
| FibG | 6.9% | 5.3% |  |  | 10.6% | 4.3% |
| Hp | 10.2% | 9.4% | 5.3% | 5.5% | 6.8% | 9.4% |
| Hx | 6.4% | 1.4% | 4.6% | 3.0% | 10.0% | 1.5% |
| IgM | 6.1% | 2.6% | 6.1% | 4.6% | 9.8% | 2.1% |
| LPSBP | 7.6% | 6.5% | 6.4% | 6.2% | 10.2% | 3.7% |
| MBL | 8.1% | 7.8% | 11.1% | 11.2% | 14.2% | 12.1% |
| MPO | 11.7% | 13.1% | 5.8% | 5.5% | 10.5% | 9.7% |
| SAA | 10.9% | 8.8% | 5.0% | 4.8% | 11.6% | 3.7% |
| Average | 8.9% | 6.7% | 6.5% | 5.9% | 10.2% | 4.5% |

Supplementary Table 2

| Subject | Total samples | Inflammation events | Samples with CRP>2-fold over baseline | Samples in events |  | Fraction samples with CRP>2-fold over baseline | Fraction samples in events |
| --- | --- | --- | --- | --- | --- | --- | --- |
| S-01 | 186 | 9 | 47 | 91 |  | 25% | 49% |
| S-04 | 250 | 9 | 78 | 109 |  | 31% | 44% |
| S-07 | 49 |  | 6 | 0 |  | 12% | 0% |
| S-10 | 284 | 11 | 128 | 67 |  | 45% | 24% |
| S-17 | 87 | 1 | 30 | 8 |  | 34% | 9% |
| S-18 | 411 | 20 | 77 | 133 |  | 19% | 32% |
| S-20 | 45 | 2 | 14 | 8 |  | 31% | 18% |
| S-22 | 210 | 6 | 45 | 34 |  | 21% | 16% |
