# Supplementary figures and images for: Precision multiparameter tracking of inflammation on timescales of hours to years using serial dried blood spots
Source: Bioanalysis. 2020 Apr 7:10.4155/bio-2019-0278. doi: 10.4155/bio-2019-0278 (PMC7372997; doi:10.4155/bio-2019-0278)

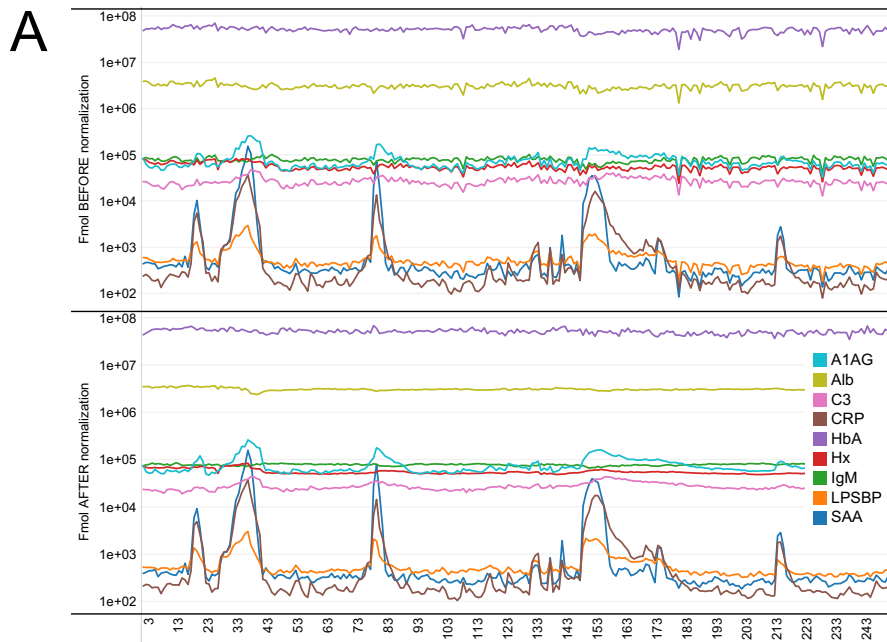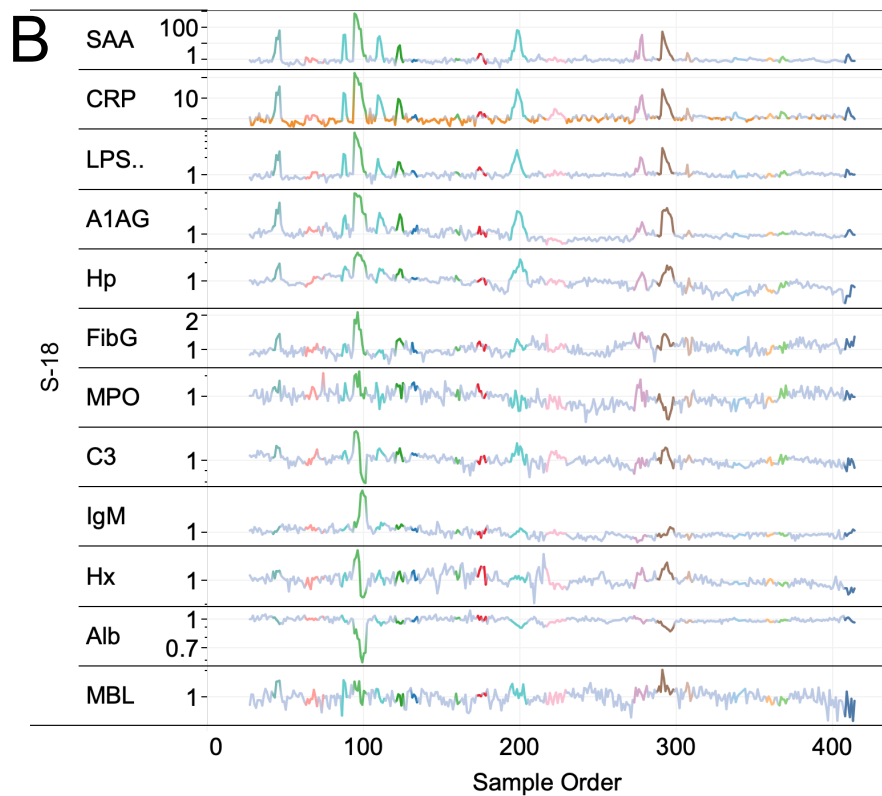

Supplementary Fig. 1

Supplement: Supplementary file 2 [file bio-2019-0278-s2.pdf]

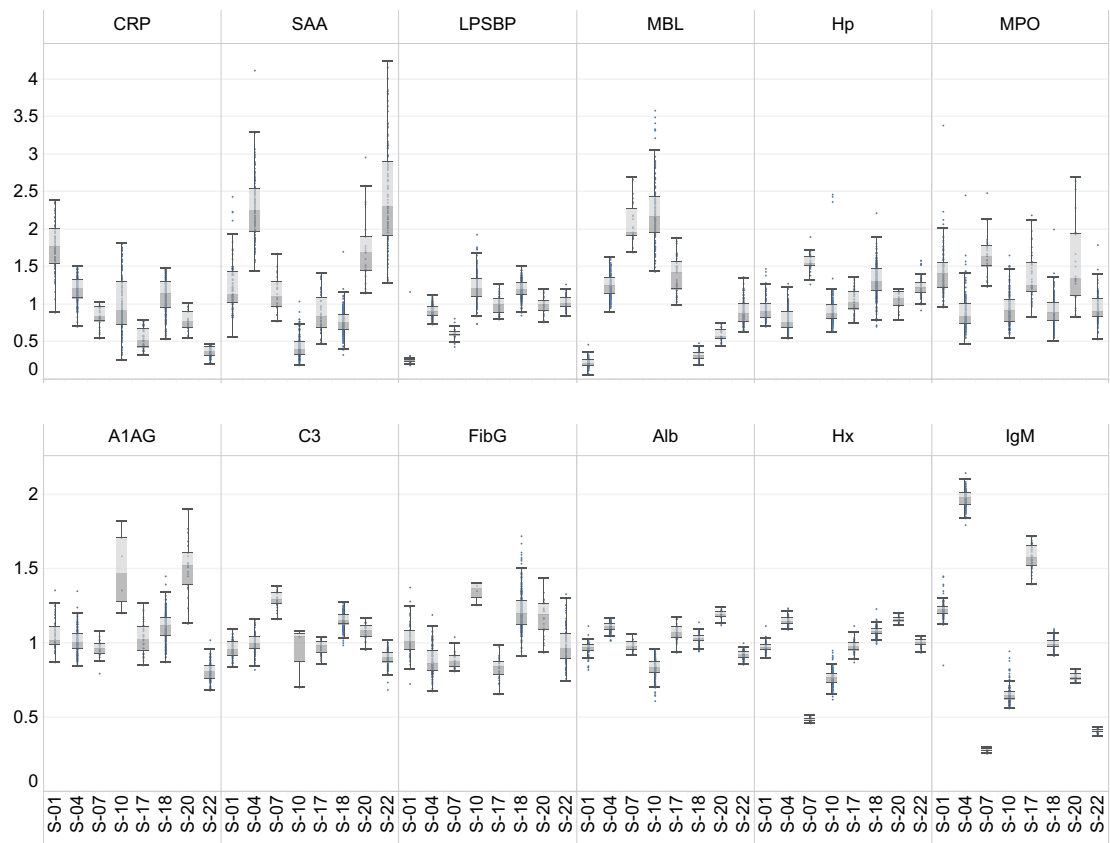

Supplementary Fig. 2

Supplement: Supplementary file 3 [file bio-2019-0278-s3.pdf]

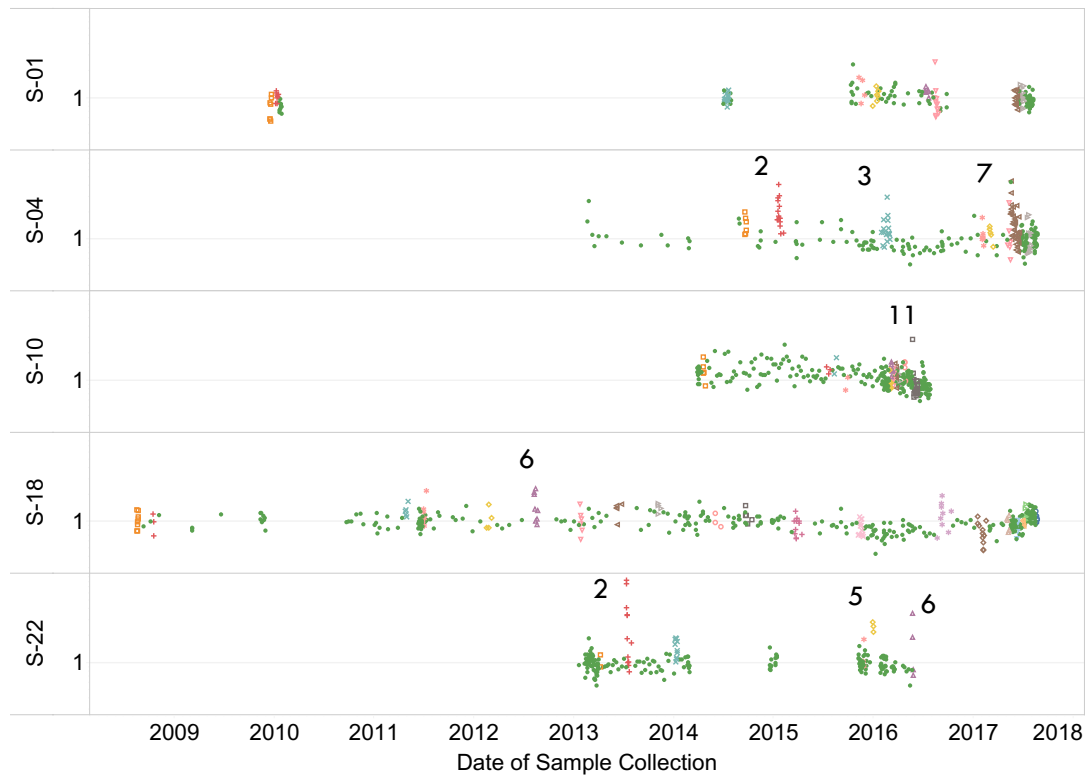

Supplementary Fig. 3

Supplement: Supplementary file 4 [file bio-2019-0278-s4.pdf]

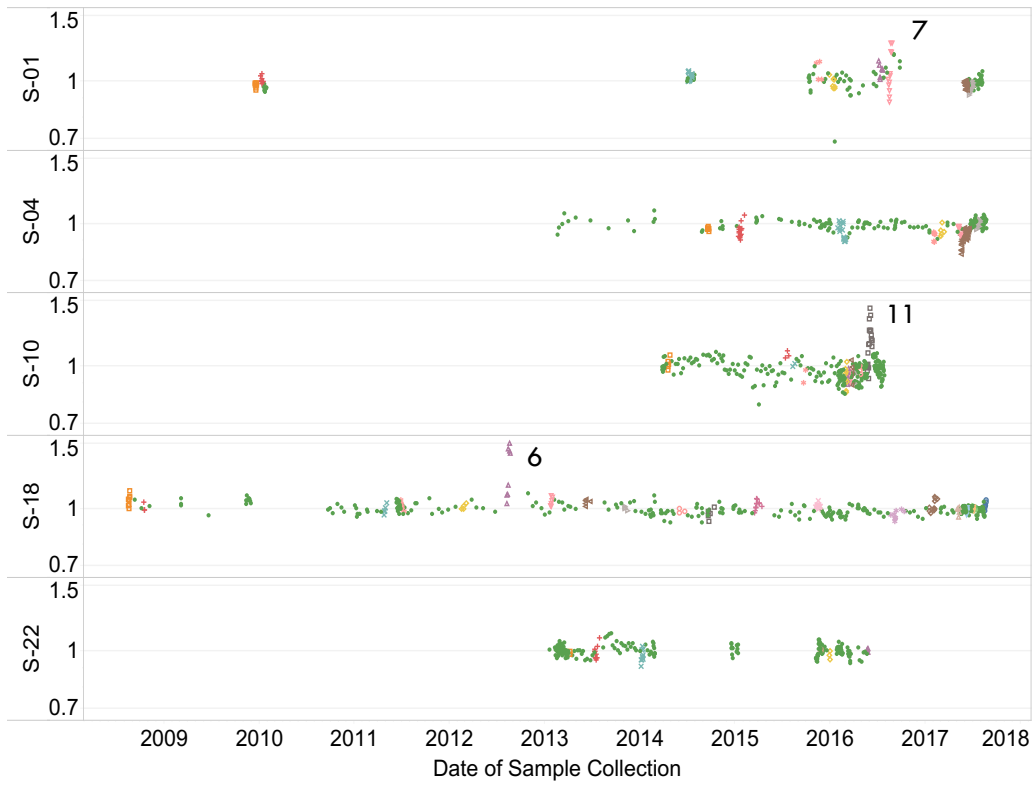

Supplementary Fig. 4

Supplement: Supplementary file 5 [file bio-2019-0278-s5.pdf]
